# Influence of Estrogen on the NSCLC Microenvironment: A Comprehensive Picture and Clinical Implications

**DOI:** 10.3389/fonc.2020.00137

**Published:** 2020-02-18

**Authors:** Tanner Smida, Tullia C. Bruno, Laura P. Stabile

**Affiliations:** ^1^Department of Immunology, University of Pittsburgh, Pittsburgh, PA, United States; ^2^UPMC Hillman Cancer Center, Pittsburgh, PA, United States; ^3^Department of Pharmacology & Chemical Biology, University of Pittsburgh, Pittsburgh, PA, United States

**Keywords:** estrogen, tumor microenvironment, NSCLC, sex disparities, immune checkpoint inhibitors, anti-tumor immunity

## Abstract

Lung cancer mortality represents the leading cause of cancer related deaths in the United States and worldwide. Almost half of these deaths occur in female patients, making lung cancer the most common cause of cancer mortality in women with a higher annual mortality rate than breast, uterine, and ovarian cancers combined. The distinct epidemiological, histological and biological presentation of non-small cell lung cancer (NSCLC) in women combined with extensive preclinical data have demonstrated that the female sex hormone β-estradiol (E2) plays an important role in NSCLC tumorigenesis, prognosis, and treatment response. Estrogen receptors are widely expressed on stromal and immune cells, and estrogen-linked signaling pathways are known to be involved in regulating the response of both the innate and adaptive immune system. Immune evasion has been recognized as a “hallmark” of cancer and immunotherapy has re-defined standard of care treatment for NSCLC. Despite these advancements, the low response rates observed in patients treated with immune checkpoint inhibitors has led to a search for mediators of immunosuppression and ways to augment the action of these agents. We focus on emerging data describing sex differences that modulate immunotherapy efficacy in NSCLC, immunosuppressive properties of E2 that lead to a pro-tumor microenvironment (TME), and the translational potential of altering the immune microenvironment by targeting the estrogen signaling pathway. E2-induced modulation affects multiple cell types within the TME, including cancer-associated fibroblasts, tumor infiltrating myeloid cells, and tumor infiltrating lymphocytes, all of which interplay with lung tumor cells via E2 and estrogen receptor engagement, ultimately shaping the TME that may, in part, be responsible for the sex-based disparities observed in NSCLC. An improved understanding of the role of the estrogen pathway in NSCLC anti-cancer immunity may lead to novel therapeutic approaches for altering the TME to improve the efficacy of immunotherapy agents.

## Introduction

The high incidence and mortality rate of lung cancer represents a global health problem. Within the United States, over 228,150 new lung cancer cases are predicted to occur in 2019 (1). Of these cases, non-small cell lung carcinoma (NSCLC) accounts for ~85% of diagnoses. Despite advances in the frontline standard of care for NSCLC, the prognosis for this disease remains poor, with an estimated overall 5-year survival rate of only 23% for all stages (1). Targeted therapies have improved outcomes for molecularly-defined NSCLC subgroups, but, for most patients, resistance to these targetable agents is inevitable. The field of immune-oncology has rapidly evolved, and immune-based therapies, including antibodies that block checkpoint signals such as programmed death 1 (PD-1) and programmed death-ligand 1 (PD-L1) have revolutionized treatment for NSCLC. Despite durable remissions and prolonged survival in a subset of patients, 80% of patients with advanced NSCLC do not respond to currently approved single checkpoint inhibitor-based immunotherapy (2, 3), and both acquired and adaptive resistance are observed (4). There is an unmet need for treatment of the many patients that do not respond to current immune modulation and a need to improve the effectiveness of current immunotherapeutic approaches in NSCLC.

There is also a growing public health concern that the NSCLC incidence and mortality in women and never smokers, the majority of whom are female, is rising. While the role of the estrogen pathway in the development of NSCLC is multi-faceted and not yet fully defined, there is sufficient data to show that it plays an important role in lung tumorigenesis, biological presentation, prognosis, and treatment response. Estrogen activity not only has pro-growth effects directly on lung tumor cells but may also influence the immune and stromal cells within the tumor microenvironment (TME), thereby contributing to immune suppression through immune checkpoint regulation. In addition, preclinical, and clinical data show that estrogen pathway antagonizing agents are beneficial for NSCLC treatment, and likely exert their effects through the ablation of estrogenic signaling not only in tumor cells but also in cellular components of the TME. Considering the heterogeneous role of estrogen in NSCLC and its influence on the immune contexture, hormonal downregulation may promote an anti-tumor immune response.

## Sex Differences In Nsclc Incidence, Presentation, and Prognosis

Over the past two decades, a substantial body of data has emerged demonstrating sex disparities in the incidence, presentation, and prognosis of NSCLC. Although smoking is the causative factor in ~80% of all lung cancer cases in both men and women, other factors such as genetic and epigenetic differences, environmental exposures, lifestyle factors, and sex hormones (both endogenous and exogenous) are known to play an important role in lung carcinogenesis and may be responsible for the observed sex disparities in lung cancer. It is now recognized that NSCLC in women presents with a distinct epidemiological, histological, biological, and prognostic footprint that suggests a role of female sex hormones in the carcinogenesis and progression of this disease.

Since the popularization of cigarettes at the end of the nineteen century, NSCLC rates in male patients have far outpaced those observed in women due to the higher levels of smoking in men. The average smoker experiences a 20-fold increase in risk compared to never smokers. However, a recent turnaround in the historical pattern of male/female lung cancer cases has been observed. Among all men and women suffering from lung cancer, up to 20% are never smokers, and ~15% are diagnosed under age 50 (5). The epidemiological trends displayed by this disease suggest that the proportion of never smoking NSCLC patients has almost doubled from 8% in the years 1990–1995 to 15% in the years 2011–2013 (10.2–22.1% [*P* < 0.001]) in women, and 6.6–8.9% [*P* = 0.006] in men) (6). While the risk of developing lung cancer has been suggested to be similar between men and women with comparable tobacco exposure (7, 8), never smokers with lung cancer are ~ 2.5 times more likely to be women than men and less likely to be current smokers (7–10). Furthermore, women are more likely to be diagnosed at a younger age compared to men. In this regard, a nationwide study of NSCLC suggested that the higher rate of NSCLC occurrence in women may be specifically attributable to the younger age groups considered in these analyses (11). The lack of a significant difference in NSCLC incidence observed between males and post-menopausal females coupled with the increased incidence in premenopausal females in comparison to these two groups points to estrogen as the causative variable. Changing demographical patterns associated with NSCLC in the United States may provide insight into this phenomenon as well. A recent analysis conducted by the North American Association of Central Cancer Registries found a disproportionate increase in incidence rates among premenopausal women when compared to their male counterparts that began to manifest in the mid-1960s in tandem with the decrease in overall lung cancer diagnoses due to a marked reduction in smoking behavior (11, 12). Alongside an overall decrease in NSCLC incidence rate from 1995 to 2014, the female to male incidence ratios increased in the 30–49 age cohort (11). This shift cannot be linked to changes in smoking behavior among women, and illustrates the urgent need to understand what is driving this trend.

Like the epidemiology, the molecular presentation of NSCLC differs by sex both by gross histology and the more granular classification by oncogenic drivers. Women are more likely to be diagnosed with adenocarcinoma (AC) histology, while men are more likely to present with squamous cell carcinoma (SCC) (9, 13). For males, the ratio of AC to SCC is ~1:1; for females it is ~2:1. Targetable driver mutations in lung ACs are more often observed in lung tumors from women than men. For example, mutations in the epidermal growth factor receptor (*EGFR*) occur with a higher frequency in women with AC of the lung than in men (14). This pathway serves as a target for small-molecule tyrosine kinase inhibitors (TKIs) such as erlotinib and osimertinib, which have become first-line treatment options for *EGFR* mutant NSCLC (15). Anaplastic lymphoma kinase (*ALK*) oncogene related translocations also occur more frequently in female NSCLC patients, presenting another well-defined drug target to clinicians (16, 17). Likewise, the rare *ROS1* rearrangements are also more common in lung ACs from women than men (18–20). Among smokers, the occurrence of *KRAS* and *p53* mutations are more common in females than males (21–23), which is likely related to an increased susceptibility of tobacco carcinogen induced DNA adducts in female patients.

Both randomized controlled trials (RCTs) and composite meta-analyses have suggested a difference in the prognosis for NSCLC between male and female patients. Women present a marked survival advantage over their male counterparts regardless of significant factors such as age, histology, and smoking status (24–26). One reason for this could be the aforementioned higher incidence of targetable somatic alterations in oncogenic pathways such as *EGFR*, which lead to more effective treatment strategies. A meta-analysis found that female NSCLC patients derive a greater benefit from *EGFR* TKIs than men (HR = 0.34; 95% CI = 0.28–0.40; *P* < 0.00001 vs. HR = 0.44; 95% CI = 0.34–0.56; *P* < 0.00001, for women and men, respectively) (27). A prognostic dichotomy among female patients based on hormonal status is observed with NSCLC as well; among women, worse survival, and the diagnosis of more advanced-stage disease is observed in premenopausal groups when compared to postmenopausal females as well as their male counterparts, adding additional support to an estrogenic role in NSCLC (28). While several studies show that female patients respond better to radiotherapy and chemotherapeutic agents, with more frequent, durable responses, and longer progression free survival (PFS) (28–31), these studies have generally only included postmenopausal women in their analyses. Even though females generally have better survival compared to men, a recent global analysis has projected that lung cancer mortality rates among women will increase by 43% from 2015 to 2030 (32), likely due to the increase in NSCLC incidence rates in this population.

Most recently, the estrogenic influence has been suggested to have an impact on the response rate to immune checkpoint inhibitors (ICIs), which have become a frontline therapy and a standard of care for many cancer subtypes, including NSCLC (33, 34). The most well-characterized immune checkpoints at this time include PD-1, PD-L1, and cytotoxic T-lymphocyte-associated protein-4 (CTLA-4) and are the main targets of these agents (35–37). PD-1 and CTLA-4 are predominately expressed on the surface of T cells (38, 39). PD-L1 is expressed on the surface of immune cells in addition to being present in a variety of tissues and tumor types, including NSCLC. The ligation of immune checkpoints such as PD-1 and CTLA-4 leads to immune response depression primarily through the inception of T cell “exhaustion,” a state marked by reduced levels of proliferation, effector function, and survival (40). The mechanism of action of all ICIs revolve around preventing the interaction of the immune checkpoints and their ligands, thus preventing the loss of a cytotoxic response to cancer cells. One major problem with the use of ICIs is low response rates, as ~ 80% of recipients with NSCLC show no benefit (41). For this reason, there is a clear need for the characterization of biomarkers that predict response as well as development of novel strategies to augment response. Tumoral PD-L1 expression was initially used for this purpose, but its predictive value has been questioned due to the lack of standardization in antibody use, threshold for positive classification and the biological heterogeneity in expression coupled with the observation that some PD-L1 negative tumors respond to ICIs, while other PD-L1 positive tumors do not (42–44). Tumor mutational burden (TMB), as quantified by the number of DNA mutations per megabase, has emerged as a potential biomarker for ICI response (42). This may be due to the correlation of TMB with neoantigen presentation by the tumor, which leads to a stronger immune response. An examination of 335 lung AC cases demonstrated that TMB was highest in males and smokers (45), which correlates with the response to ICIs observed in NSCLC patients. Interestingly, a recent study found that use of TMB to predict ICI response was poor for male NSCLC patients, but was a strong predictor for ICI response in females (46).

Several recent studies showed that female sex is a negative predictor for response rates to ICIs (27, 47–50), and resistance is often observed in this group (4). In a meta-analysis of 20 Phase II and III RCTs that included over 11,000 advanced cancer patients, including NSCLC, a statistically significant difference in efficacy between male and female patients treated with CTLA-4 or PD-1/PDL-1 blocking antibodies compared to standard of care treatment was observed (*P* = 0.0019) (47). While both male and female patients showed reduced risk of death with ICI, the benefit for females was not as strong as for males. For male patients treated with ICIs, the pooled overall survival hazard ratio (OS-HR) was 0.72 (95% CI = 0.65–0.79) while for females, the pooled OS-HR was 0.86 (95% CI = 0.79–0.93) (47). A second meta-analysis from this same group was performed focusing on advanced NSCLC patients only, which included all RCTs evaluating an anti-PD-1/PD-L1 agent alone or combined with chemotherapy (50). Male patients that were treated with anti-PD-1 alone experienced a reduction in their risk of death compared to men treated with chemotherapeutic agents (OS-HR = 0.78, 95% CI = 0.60–1.0), while there was no difference in risk between anti-PD-1 alone or standard chemotherapy in females (OS-HR = 0.97, 95% CI = 0.79–1.19) (50). Conversely, an analysis of anti-PD-1/PD-L1 agents plus chemotherapy in comparison to chemotherapy alone in lung cancer patients found that women had a large OS advantage with combined anti-PD-1/PD-L1 with chemotherapy vs. chemotherapy alone, with a much smaller benefit observed in the male patients (OS-HR = 0.44 vs. 0.76 for female vs. male, respectively) (50). These results were confirmed in an additional analysis in lung cancer patients where OS was favorable in male patients receiving ICIs compared to chemotherapy (HR = 0.76; 95% CI = 0.68–0.86) but not in females (HR = 1.03; 95% CI = 0.89–1.03) (27). Another meta-analysis of 8 RCTs aimed at determining predictors of clinical benefit from ICIs in metastatic NSCLC reported a significant increase in PFS for males, smokers, and PD-L1 positive subgroups treated with ICIs compared to chemotherapy (48). Female and PD-L1 negative patients showed similar benefit with ICIs and chemotherapy (48). These findings underscore the complexity of the sex-based influences on the immune system and response to treatment. One potential mechanism for the increased benefit from combined chemotherapy and ICI treatment in females is the ability of chemotherapy to increase the mutational burden and tumor antigen release into the microenvironment, leading to mitigation of immunosuppressive effects.

## Evidence For Estrogen Signaling in Lung Cancer

Preclinical *in vitro* and *in vivo* studies prompted by the epidemiological sex disparities have provided additional support linking estrogen signaling to lung tumorigenesis. These studies led to the completion of several clinical trials testing the pure antiestrogen fulvestrant in NSCLC patients. These studies are summarized below.

### Preclinical Studies

Histological surveys of patient tissue have shown that NSCLC is known to be a primarily estrogen receptor (ER) positive tumor type and expresses aromatase, the enzyme that catalyzes the final step of estrogen synthesis (51, 52). Of the two isoforms of this receptor (ERα and ERβ), ERβ is dominantly expressed (~ 80–90% of cases) in NSCLC patient tumor specimens and cell lines derived from both males and females, while ERα expression is generally low (3, 52–56). Furthermore, the biological effects of estrogen are known to be primarily mediated by ERβ (52, 53, 56). While the G-protein coupled estrogen receptor (GPER/GPR30) is also found in NSCLC (57), this review will only focus on the classical ERs.

Preclinical studies of the effect of β-estradiol (E2), the main biologically active female sex hormone, in NSCLC have demonstrated marked proliferative effects that occur through both genomic transcriptional regulation as well as through indirect non-genomic mechanisms (53, 56, 58, 59). This ER modulated influence on cells is actualized by cell cycle regulation through ERK and EGFR signaling, the cAMP, MAPK, and AKT pathways, and the promotion of c-myc and cyclin D expression (60, 61). E2 has also been shown to promote angiogenesis in NSCLC cell lines through the secretion of vascular endothelial growth factor (VEGF) (62), providing a link between estrogen and a well-defined “hallmark” of cancer (63). An interaction between the ER pathway and the fibroblast growth factor receptor (FGFR) pathway has also been observed both in a murine model of lung cancer and in NSCLC patient tissue (64).

*In vivo* models of lung cancer have demonstrated the utility of targeting the ER pathway therapeutically. In xenograft models of lung cancer, the administration of the antiestrogen fulvestrant, which targets both ERα and ERβ, resulted in suppression of tumor growth (53, 64, 65). Fulvestrant was also shown to prevent lung tumorigenesis in mice exposed to the tobacco carcinogen, 4-(methylnitrosoamino)-1-(3-pyridyl)-1-butanone (NNK), suggesting possible value for the use of antiestrogens as protective agents against smoking induced lung cancer (66). Similarly, aromatase inhibitors, including anastrozole and exemestane, were also effective at inhibiting tumor growth and tumor incidence in multiple NSCLC murine models (66–68). Furthermore, the combinatorial use of both fulvestrant and anastrozole demonstrated a synergistic, inhibitory effect on both initiation, and progression of lung tumors in the NNK lung cancer model (66). Based on the cross-talk between the estrogen and growth factor pathways described above, estrogen targeting agents have also been evaluated in a preclinical context in combination with EGFR, FGFR and VEGFR TKIs, demonstrating increased anti-tumor effects with combinatorial treatments compared to single agents (59, 62, 64, 65).

### Clinical Studies

We summarize below the established correlation between pharmacologic hormonal modulation (though hormone replacement therapy (HRT) or antiestrogen treatment in breast cancer patient cohorts) and NSCLC incidence, the prognostic value of hormonal markers in NSCLC patient tumors, and completed or ongoing clinical trials testing agents that target estrogen signaling for NSCLC patients.

Results from the Women's Health Initiative (WHI) randomized, double blind controlled trial conducted with the goal of determining the effects of hormone replacement therapy (HRT) on postmenopausal women (*n* = 10,739) who were prescribed estrogen and progesterone (E+P) to mitigate the symptoms of menopause, found that the women taking E+P had an increase in lung cancer mortality (69). While there was also a trend in increased NSCLC incidence with HRT use in this study, it was not statistically significant. This may be a result of the limited follow-up time available at the time of analysis (mean 5.6 years of intervention + 2.4 years of follow-up) (69). Additional analysis of the data from the WHI trial demonstrated that the risk of death from lung cancer during E+P use was attenuated after the discontinuation of combinatorial hormone therapy (70). A subsequent observational study of a prospective cohort of 36,588 women as part of the Vitamins and Lifestyle (VITAL) Study demonstrated that an increased incidence of lung cancer for women treated with E+P was present in a duration-dependent manner, with an ~50% increased risk for use lasting a decade or more (71).

Another facet of this paradigm is the development of primary lung tumors in patients after hormone modulation has been used as part of a prior chemotherapeutic treatment regimen. It has been demonstrated in multiple cohorts that breast cancer patients treated with antiestrogens were less likely to develop a subsequent primary lung tumor later in life and better survival rates were observed among those that do develop NSCLC (72–76). Interestingly, the highest levels of second primary lung cancer diagnosis among breast cancer patients were present in the triple-negative breast cancer cohort (77). As these patients were not exposed to estrogen pathway modulation as a treatment modality, this observation is consistent with a role for estrogen in NSCLC.

Components of the estrogen signaling pathway have been shown to demonstrate prognostic significance in NSCLC patients. The expression of ERβ has been reported alternatively as a favorable and unfavorable prognostic marker for survival in NSCLC patients and therefore remains controversial. This may be due to a lack of standardization of the scoring systems, staining protocols, and antibody epitopes used to evaluate this biomarker (52). In addition, consideration of nuclear vs. cytoplasmic expression has proved to be an important factor. Some studies of cytoplasmic ERβ-1 overexpression show that this subtype serves as an independent negative prognostic factor for NSCLC, as well as an indicator of aggressive tumors (52, 78–80). This data suggests that non-genomic signaling by cytoplasmic ERβ has important clinical implications and warrants further study in the context of NSCLC. Although found much less frequently in NSCLC, the relationship between ERα and NSCLC prognosis is inconsistent. Some studies found a positive association with ERα and survival (81–83), while others have shown either no association with survival (52) or that high expression correlated with poor survival (84). In addition to the association of hormonal pathway proteins with survival in NSCLC, the level of E2 present in the TME has been correlated with NSCLC progression and survival. A mass spectroscopy-based analysis of 59 NSCLC patients determined that intratumoral E2 levels in NSCLC tissue were 2.2-fold higher than basal levels in non-neoplastic lung tissue (*P* = 0.0002) (85). The elevated E2 concentration was positively correlated with aromatase expression in these tissues (*P* = 0.01), suggesting local production of E2 by aromatase. The concentration of E2 was also correlated with tumor size (*P* = 0.04) in ERβ positive NSCLC (85). High aromatase expression has been demonstrated to be a poor predictor of survival in both sexes in early stage NSCLC patients. When the population considered in this analysis was subdivided by sex and age, it was determined that the value of aromatase as a predictor of survival was most pronounced in women ≥ 65 years of age (*P* = 0.006), with 79% of women in the low aromatase group surviving 5 years post-surgery as opposed to only 49% 5 year survival observed in the population with high aromatase expression (86). Multiple studies that examined ERα, ERβ and aromatase expression in NSCLC found that the combination of ERβ and aromatase was a stronger predictor of poor survival than ERβ alone in both men and women (52, 79). Circulating E2 in patient serum has also been indicated as a systemic biomarker for NSCLC progression. In a study of three NSCLC cohorts, a significant correlation (*P* < 0.001) was observed between worse survival and high circulating E2 serum levels, independent of gender and HRT usage (87). DNA polymorphisms in the estrogen biosynthesis pathway that affected serum E2 or tumor ERα expression were also associated with lung cancer survival in this study (87).

The Prediction Analysis of Microarray 50 (PAM50) gene panel, a prognostic tool in ER+ breast cancer (88), also showed prognostic value in NSCLC cohorts (89). Seven of the 50 genes from this panel were identified to have the strongest association with both disease-free survival (DFS) and PFS in NSCLC including *MYC, MIA, FGFR4, CXXC5, GRB7, FOXC1*, and *PGR*, and were retained in cross-validation. Pathway analysis predicted that this 7-gene signature comprised one interacting network with the ER, HER2, and HER3 pathways as the strongest regulators (89). Further studies demonstrate that the combination of fulvestrant with the pan-EGFR inhibitor dacomitinib in NSCLC xenograft models was able to completely reverse the 7-gene signature at both the mRNA and the protein level, in addition to showing synergistic antitumor effects (65). Importantly, these effects from the combination of fulvestrant and dacomitinib were observed in *EGFR* wild-type and *KRAS* mutant, as well as *EGFR* mutant NSCLC models.

Several clinical trials have evaluated fulvestrant treatment in combination with EGFR TKIs in NSCLC based on the preclinical discovery of crosstalk between these two pathways. These combination treatments were shown to be well-tolerated by NSCLC patients and demonstrated a moderate increase in responsiveness and OS compared to the single treatment arm alone (90, 91). A component of the Phase I study of gefitinib combined with fulvestrant evaluated the correlative relationship between tumoral ERα and ERβ protein expression and response to treatment. The patients that had >60% ERβ tumoral expression had an OS of 65.5 weeks, while the patients that had <60% ERβ tumoral expression experienced an OS of 21 weeks. No association of ERα with outcomes was found (90). A subsequent Phase II trial of erlotinib with fulvestrant vs. erlotinib alone demonstrated that the increased PFS and OS observed in the combination treatment arm was due to effects exclusively observed in *EGFR* wild type patients. No significant effects on PFS or OS were observed in the *EGFR* mutant group. *EGFR* wild type patients compared to mutant patients were also more likely to be hormone receptor (ERα or progesterone receptor) positive (50 vs. 9.1%) (91). The preclinical study described above combining fulvestrant with the pan-EGFR inhibitor dacomitinib (65) suggests that the modest improvement in response that was found in these early phase studies evaluating fulvestrant combined with the first generation EGFR TKIs gefitinib or erlotinib may be improved with a pan-EGFR inhibitor that also targets HER2/HER3 and warrants evaluation in a clinical trial. A Phase II European-led clinical trial known as the Lung cancer in women treated with Anti-estrogens anD Inhibitors of EGFR (LADIE) has recently been completed and results are pending (NCT00100854). This trial evaluated fulvestrant plus erlotinib in the second- or third-line setting in women with *EGFR* wild type tumors or fulvestrant plus gefitinib in the first- or second-line setting in women with *EGFR* mutations. Two clinical trials evaluating the effects of aromatase inhibitors in NSCLC are currently active. A Phase II trial (NCT02666105) is evaluating exemestane in post-menopausal women with advanced NSCLC after disease progression on an ICI while the other Phase I trial (NCT01664754) is evaluating the safety and tolerability of the combination of exemestane with pemetrexed disodium and carboplatin in post-menopausal women with Stage IV NSCLC.

## Estrogen Modulates the Lung Cancer TME

**Figure 1:**
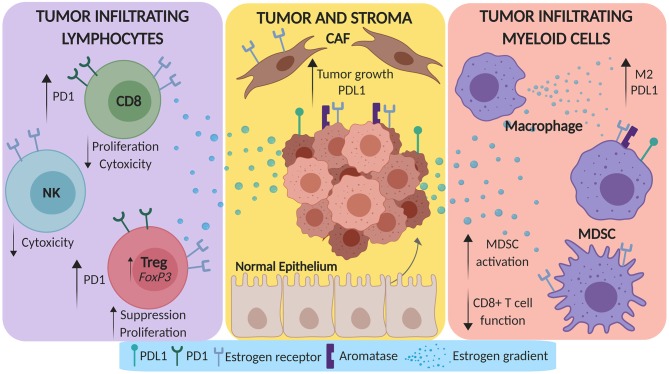
Estrogen promotes a pro-tumorigenic microenvironment in lung cancer. Estrogen can be secreted by both tumor cells and tumor associated macrophages in the tumor microenvironment (TME). This estrogen gradient can amplify tumor growth and PD-L1 expression by tumor cells via direct modulation of cancer associated fibroblasts (CAFs). Beyond the stroma, estrogen can skew myeloid cells in the TME toward M2 differentiation and can expand myeloid derived suppressor cells (MDSCs). Lastly, estrogen can dampen the CD8^+^ TIL response via upregulation of PD-1, decrease the cytotoxicity of NK cells, and increase the suppressive function of T-regulatory cells (Tregs).

The direct interplay between tumor, stroma and pro- and anti-tumor immune cells molds the complete lung TME. There is substantial evidence that suggests estrogen has a pro-tumorigenic role in the TME, which can be summarized by the modulation of stromal cells, tumor infiltrating myeloid cells, and tumor infiltrating lymphocytes as depicted in Figure 1. While some studies in lung cancer preclinical models and patients have been completed to assess this modulation, the majority of these affects have been more comprehensively studied in canonical hormone-dependent solid tumors due to the clear role of estrogen in these incidences.

### Estrogen Drives Pro-tumorigenic Stromal Cells in the TME

It is clear that E2 has a direct effect on the biology of lung tumor cells. Within the contexture of the TME, these effects are magnified by the synergistic involvement of stroma and immune cells. The stroma is defined as a network of connective tissues that secretes supportive extracellular matrix and consists primarily of fibroblasts and epithelial cells. In general, experimental studies have demonstrated that the expression of ERα on stromal cells within the TME mediates the pro-tumor effects of E2. For example, a murine model of breast cancer demonstrated that mice seeded with ERα deficient tumor cell lines continued to demonstrate accelerated tumor growth and modification of the tumor vasculature in the presence of E2. This was explored further through the use of genetically modified ERα^−/−^ mice (92). The pro-tumor effect of E2 addition in mice with ERα negative tumors was completely abrogated in the ERα knockout mice, demonstrating that ERα expression in the stroma is necessary for estrogen mediated effects on the TME (92). This effect was not observed in ERβ knockout mice, further implicating ERα as the main effector of the observed pro-tumor estrogenic effects.

Within the stroma, fibroblasts represent a heterogeneous population with phenotypic plasticity. They secrete large amounts of soluble factors that regulate tissue homeostasis as well as participating in wound healing and senescence (93). In solid tumors, normal fibroblasts differentiate to cancer associated fibroblasts (CAFs), which can modify the behavior of surrounding stromal and tumor cells (94). CAFs are the most prominent non-cancer cell in many solid tumors (95) and because they are normally in a constitutively active state, they are often large contributors to key interactions within the TME. It has been established that CAFs can remodel the TME via activation of ER, and evidence to support this has been outlined in breast, prostate, and cervical cancer.

One study on CAFs utilized nuclear receptor arrays to compare gene expression profiles between primary CAFs and normal fibroblasts from primary breast cancer tissue, and ultimately reported the presence of ERα expression in fibroblasts from these samples (96). Thus, expression of ER is elevated on CAFs and this often pairs with downstream effects on the E2 responsive gene, liver receptor homolog-1 (*LRH-1*) (96), which is a direct transcriptional regulator of the aromatase encoding gene, *CYP19A1* (97–99). Subsequently, the co-expression of *LRH-1* with aromatase suggests a paracrine mechanism of E2 synthesis and ER-mediated oncogenesis in the breast TME (100). In endometrial cancer, CAFs can also express both ERs, which promotes tumor cell proliferation. Further, endometrial CAFs have been shown to induce *in vitro* tumor cell proliferation through activation of the PI3K and MAPK signaling networks, well-characterized pathways present in breast and lung cancer that are known to be modulated by estrogenic signaling (59, 101–103). In cervical cancer, E2 has been implicated as a cofactor in human papillomavirus (HPV)-mediated disease in both preclinical models and women with cervical cancer. Specifically, primary CAFs sorted from mammary, pancreatic, and a K14 HPV16 E6/E7 mouse model of skin were found to express a pro-inflammatory gene signature; however, this effect was not pronounced in a K14 HPV16 E6/E7 model of cervical cancer (104). This ultimately suggests that a key portion of the TME milieu is distinct. In this case, the transgenic mouse model for cervical carcinogenesis is E2 dependent, operating through ERα. In addition, paracrine mechanisms involving stromal ERα signaling are necessary for oncogenesis (105–107). To supplement these murine studies, human cervical tumors have been shown to express ERα abundantly on activated fibroblasts (108, 109). The functional consequence of ERα on cervical cancer CAFs was further elaborated in a study where *ex vivo* CAFs were analyzed for dominant gene expression patterns. These studies demonstrated that CAFs had pro-tumorigenic and pro-inflammatory signaling and that these prominent functions were under the control of ERα signaling (110).

Despite the above reports on ER+ CAFs, there are very few studies that further dissect the heterogeneity of CAFs. However, a new study in breast cancer demonstrates the role of estrogen on specific subsets of CAFs. Specifically, the study highlights two subtypes of CAFs, CD146^+^, and CD146^−^ CAFs. CD146^+^ CAFs maintain ER expression in ER+ breast cancer cells and sustains estrogen-dependent proliferation and sensitivity to tamoxifen (111). These data indicate that CAF composition can contribute to treatment response and patient outcomes in ER+ breast cancer, ultimately pointing to the importance of CAF heterogeneity when developing new therapeutics.

CAFs have also been described to have a differential role on the metastatic tumor environment. There have been studies suggesting that CAFs contribute to the metastatic process (112). In particular, studies have suggested that CAFs induce the mesenchymal to epithelial transition, ultimately facilitating the metastatic colonization of primary tumor cells (113). However, studies in prostate cancer have described a different role for ER+ CAFs. These studies demonstrated that ER+ CAFs were unable to recruit macrophages when the chemokine CCL5 was inhibited, ultimately reducing the invasiveness of prostate cancer tumor cells (114). Further, microRNAs are differentially regulated by E2 in primary vs. metastatic breast cancer CAFS, which may offer further insight into differences in downstream function (115).

### Estrogen Modulates Tumor Infiltrating Myeloid Cells

Macrophages comprise a large fraction of the myeloid compartment in the TME and have been implicated in pro-tumor activity (116, 117). Conventionally, the differing functional states of macrophages defines “classically activated” pro-inflammatory macrophages as M1 and “alternately activated” anti-inflammatory macrophages as M2 (118). Physiologically, macrophages exist on a continuum between these two functional states, rather than as a clear M1/M2 dichotomy. Macrophages express both ERs in addition to aromatase, and both alveolar and bone marrow derived macrophages from female mice show increased M2 markers (119) compared to males. In a mouse model of lung inflammation, ovariectomized mice showed reduced M2 polarization in alveolar macrophages while E2 supplementation enhanced IL-4 mediated M2 polarization (119–122).

Tumor associated macrophages (TAMs) mainly resemble M2-like phenotypes and suppress antitumor immune responses, along with enhancing tumor angiogenesis, and migration (123). TAMs have been evaluated with outcome of cancer patients, with contradictory results (124–128). In breast tumors, ER status has been shown to be an important determinant of association of TAM expression with outcome (129, 130). High levels of intratumoral TAM infiltration was a robust marker of poor survival in ER and/or progesterone receptor positive tumors (*P* = 0.015). The number of stromal TAMs was also predictive, but to a lesser extent (*P* = 0.045) (129). In this study, TAM infiltration did not show prognostic value in the hormone receptor negative group, suggesting a role of ER in the mediation of the protumor effects by these myeloid cells (129).

TAM infiltration is associated with the expression of the chemoattractants CCL2 and CCL5, which are influenced by E2 in breast cancer. In murine breast tumor models, E2 promoted tumoral M2 macrophage infiltration through increased CCL2 and CCL5, while the antiestrogen tamoxifen reversed this effect and exhibited M1 TAMs instead (120). Observational studies of postmenopausal patients with breast cancer treated with tamoxifen also displayed reduced CCL2/CCL5 (120). The discovery of aromatase positive macrophages and adipocytes in breast tissue have been shown as a contributing mechanism to obesity induced breast cancer in post-menopausal women (131). In an ovarian cancer murine model, tumors from mice treated with E2 had a significantly higher TAM density compared to tumors grown in ovariectomized mice, which translated to enhanced TAM infiltrate in ovarian cancer tissue specimens from premenopausal relative to postmenopausal women (122).

*In vivo* studies using the tobacco carcinogen NNK induced lung cancer model demonstrated that administration of E2 in mice increased pulmonary TAM infiltration (67). In lung preneoplasias that developed after NNK plus E2 treatment, a significant increase in inflammatory markers and VEGF, an E2 reponsive gene and regulator of macrophage recruitment and the M1/M2 macropahge switch, was found compared to control (67). Conversely, in NNK treated mice that received the aromatase inhibitor anastrozole, a significant reduction in pulmonary TAMs was found, which were found to be aromatase and ERβ positive (67). Addition of a non-steroidal anti-inflammatory agent to anastrozole was shown to further reduce TAM recruitment in this model (67). A limitation to the studies in the NNK model of lung cancer is that TAM expression was assessed through IHC and M1/M2 phenotypes were not distinguished. Expression of aromatase by TAMs has also been demonstrated in patient NSCLC tumors (52). An additional study showed that M2 polarization of human monocyte derived macrophages by lung tumor conditioned media was prevented by the estrogen blocker resveratrol, an effect that was also observed in a lung tumor mouse model along with decreased tumor growth (132).

Due in part to the mix of signaling molecules present in the TME, myelopoiesis is disregulated and leads to the generation of a heterogenous group of immunosuppressive cell types known as myeloid derived suppressor cells (MDSCs) (133). MDSCs promote numerous pro-tumor effects in the TME, including angiogenesis, metastasis, and the suppression of T cell response (116). All of the cell types included under the MSDC umbrella express ERs, making the estrogen pathway a possible regulator of activity within this compartment. In estrogen-insensitive ovarian cancer models, estrogen promoted tumor growth by mobilizing and enhancing the inhibitory capacity of ER expressing MDSCs while estrogen depletion had the opposite effects (134, 135). Mechanistically, this occurs through estrogen induced STAT3 signaling in bone marrow precursors by transcriptional upregulation of JAK and SRC activity, culminating in increased MDSCs in tumor bearing mice. A recent study reported a similar mechanism of E2-induced MDSC expansion in preclinical breast cancer models, and that fulvestrant and newly developed selective ER downregulators (SERDs) interact with ER expressing immune populations, including MDSCs, which can reverse the E2 effects on MDSCs (136). Furthermore, combining an estrogen antagonist with anti-PD-L1 antibody treatment in murine 4T1 triple negative breast cancer cells implanted into the mammary glands of mice showed an enhanced reduction in tumor growth compared to antiestrogen treatment alone, while anti-PD-L1 alone had no effect (136). These studies have important implications for the consideration of estrogenic effects that are completely independent of tumoral ER status and a role for estrogen antagonists regardless of the ER status of the tumor. An MDSC focused analysis of 306 Stage IIB-IVA cervical cancer patients demonstrated that pregnant patients exhibiting high levels of serum E2 also had high levels of mobilized MDSCs (137). This observation was further evaluated in pregnant and non-pregnant mouse models of breast and cervical cancers. In this regard, E2 promoted the growth of ER negative cervical and breast tumor xenografts in both pregnant and non-pregnant mice by inducing MDSCs (137). Moreover, addition of an anti-Gr-1 neutralizing antibody prevented E2 induction of MDSCs and attenuated tumor growth in these models (137). Together these studies provide evidence of regulation of MDSC biology by E2 in multiple solid tumor models, however this has been largely unexplored in lung cancer.

### Estrogen Regulates Pro-tumor Infiltrating Lymphocytes

Tumor infiltrating lymphocytes (TIL), in particular CD8^+^ TIL, comprise an important part of the anti-tumor response within the TME. In fact, CD8^+^ TIL have been correlated with increased survival in cancer patients, including in NSCLC (138–142). A study conducted on breast cancer patients applied an *in-silico* machine learning approach to analyzing immune cell infiltration in the TME. It demonstrated that an inverse relationship exists between activity in the ER pathway and the infiltration of B cells, cytotoxic T lymphocytes (CD8^+^ TIL), Th1 and Th2 cells (143). Overall, estrogen inhibits lymphocyte infiltration into tumors, which is evidenced both by ERα and ERβ expression on CD8^+^ and CD4^+^ T cells and the increase of T and B cell infiltration into primary tumors when ER-related molecules (i.e., aromatase) are targeted (143). However, few studies have focused on the direct impact of estrogen on effector T cell function. One study outlines the effects of estrogen on the Th1-Th2 balance of CD4^+^ TIL. It demonstrated an increased Th2 response in animal and human models with elevated levels of estrogen (144), which results in an immunosuppressive TME via secretion of IL4 and IL13 (145). Further, aside from the indirect effects via modulation of MDSC populations (outlined above), there has only been one additional study evaluating the effects of estrogen on the cytotoxic function of CD8^+^ TIL. Specifically, ERα^+^ tumor cells from liver and breast cancer were treated with E2, which subsequently caused an upregulation of Granzyme B proteinase inhibitor-9 (PI9) (146). Increased PI9 would decrease the function of the cytolytic molecule, and ultimately, would cause immunosuppression of the CD8^+^ TIL response. While this elucidates the potential impact of estrogen on CD8^+^ TIL function, it is also an indirect mechanism.

In addition to decreasing the function of CD8^+^ TIL, estrogen can also mediate the function of regulatory T cells (Tregs). In the TME, Tregs secrete immunosuppressive cytokines and inhibit CD8^+^ TIL expansion (147, 148). Clinically, the presence of FOXP3^+^ Tregs has been used to predict high risk patients and often correlates with worse overall survival (149). To expand upon these findings, two different studies have demonstrated direct modulation of Tregs by estrogen. One trial evaluated the effects of aromatase inhibitor in breast cancer patients, which specifically pointed to a modulation of Tregs as FOXP3^+^ cells were decreased in all patients (150). In addition, a complimentary study demonstrated the effects of estrogen signaling in the context of anti-PD-1 immunotherapy. Estrogen treatment reduced Treg suppressive function, and this effect was reversed with an ERα-selective inhibitor (148). In addition to these clinical studies, physiological doses of E2 administered to immunocompetent ovariectomized mice have been shown to cause increased proliferation and levels of *FOXP3* expression in CD4^+^CD25^+^ Tregs (147, 151). Further, E2-treated CD4^+^CD25^+^ Tregs had increased suppressive capacity both *in vivo* and *in vitro*. Although these studies indicated that estrogen can induce a Treg phenotype (151), E2 concentrations in the TME are commonly dysregulated and could often be much higher than physiological, thus, further research toward this question should be completed. In cervical cancer, FOXP3 levels were reduced and suppressive function of Tregs was minimized in the presence of an ERα antagonist. These studies also found a complex of ERα, E2 and FOXP3 as well as ERα occupancy at the FOXP3 promoter (152). These data suggest that estrogenic signaling directly regulates Treg suppressive function through FOXP3.

Natural killer (NK) cells are an innate lymphocyte population expressing ERα and ERβ (153). NK cells exert their cytotoxic effects without a need for prior antigen exposure (154) and are known to play a role in both immune surveillance and tumor metastasis (153–155). Exposure of NK cells to E2 results in a reduction of effector function in both murine (156–161) and human (162) models as quantified by assays of direct lytic activity. Estrogen targeting agents have demonstrated the opposite effects on NK cell activity. For example, tamoxifen has been shown to sensitize ER-negative YAC-1 murine lymphoma cells to NK cell mediated lysis, supporting the potential therapeutic benefit of antiestrogens for patients with ER negative tumors (161). Mechanistically, tamoxifen-mediated augmentation of NK cell activity on human ovarian tumor cells and the human erythroleukemia cell line K562 involved both the Fas/FasL and perforin/granzyme pathways (163). A separate study in MCF-7 breast cancer cells demonstrated that E2 levels correlated with increased ER-mediated expression of proteinase inhibitor-9 (PI-9), the only known intracellular inhibitor of the granzyme B pathway, while PI-9 inhibition blocked the protective effect of E2 against NK-mediated apoptosis (164). The PI-9 mediated protective effect of estrogen was also observed in human liver cells that were challenged by the human NK cell line YT (146). Lastly, a quantitative high throughput screening assay conducted on a pharmaceutical library of > 2,000 clinically approved agents identified fulvestrant as a top enhancer of sensitivity of mesenchymal-like lung carcinoma cells to cytotoxic effects of antigen-specific T cells and NK effector cells, defining a role of estrogen signaling in promoting tumor resistance to immune-mediated cytotoxicity in lung cancer (165).

In addition to the above studies, an inverse correlation was reported between E2 and NK cytotoxic activity in post-menopausal women taking HRT (166, 167). NK cell lytic activity was measured in the peripheral blood from three groups of post-menopausal women before and after 3 weeks of receiving either daily oral estrogen valerate, transcutaneous estradiol, or no hormone substitution as well as blood from 20 untreated pre-menopausal women (167). Cytotoxic NK activity was elevated in the group of control post-menopausal women in comparison to the pre-menopausal cohort, while both HRT treated groups had decreased NK activity (167). A similar study evaluating the NK cell activity before and after E+P HRT reported significantly decreased IL-2 (*p* = 0.0092) and IFN-γ (*p* = 0.0017) secretion, in addition to reduced NK activity (*p* = 0.0026) after treatment (166). Conversely, post-menopausal breast cancer patients receiving tamoxifen treatment demonstrated significantly increased NK activity after only 1 month of treatment (168).

Hormonal regulation has also been linked to the metastatic role of NK cells (159, 169). Murine models of fibrosarcoma and lymphoma demonstrated that NK-mediated cytotoxicity decreased upon E2 exposure, resulting in increased incidence of lung metastases (*p* < 0.005) (159). In a separate study, rats injected via the tail vein with a syngeneic ER-negative mammary adenocarcinoma line had elevated lung metastases during the pro-estrus and estrous periods of high circulating E2 as well as in ovariectomized rats exposed to exogenous E2, an effect caused by altered NK cell number and activity (169).

## Immune Checkpoint Modulation

Expression of the key immune checkpoint proteins, PD-1 and PD-L1, have been shown to be increased by E2. In ERα positive endometrial and breast cancer cell lines, E2 increased PD-L1 protein expression in both a dose- and time-dependent manner via the phosphoinositide 3 kinase (PI3K/Akt) pathway and post-transcriptional PD-L1 mRNA stabilization (170). E2 also decreased IFN-γ and IL-2 expression in T cells co-cultured with tumor cells, suggesting E2-induced inhibition of T cell function (170). The suppressive action of T cells is also dependent on PD-1 expression in Tregs, which is also increased by E2 (171). E2 also increased PD-L1 expression on T cells from the reproductive tract tissues (172, 173). This provides another mechanism by which the adaptive component of the TME can have an estrogen dependent effect on the therapeutic efficacy of ICIs. Several studies examining PD-L1 protein expression in NSCLC showed no difference in PD-L1 expression in men and women (174, 175); however, other cancer types such as oral squamous cell carcinoma has shown more frequent PD-L1 expression in tumors from females than males (176).

Estrogen modulators have been shown to boost the efficacy of ICIs in preclinical breast cancer models, leading to enhanced anti-tumor effects through interaction with ER+ immune subtypes in the TME (136). Searches for new agents to boost cytotoxic immune response and to reduce the resistance observed with ICIs are also pointing to the effectiveness of estrogen modulation (165). The screening assay mentioned above that identified fulvestrant as the top drug to increase the sensitivity of lung cancer cells to both immune and chemotherapy mediated lysis (165). Importantly, a number of clinical trials are currently underway with the goal of evaluating the added benefit of estrogen modulating drugs to ICIs in the context of breast cancer (NCT02997995, NCT02778685, NCT03280563, NCT02990845, NCT02971748, NCT02648477, NCT02971761, NCT02997995) (177). These trials all combine ICIs targeting CTLA-4, PD-1, or PD-L1 with agents that target the estrogen pathway such as fulvestrant or exemestane. Given this information, it is evident that estrogen plays a role in the composition and functionality of the TME, specifically in regards to cell mediated immunity. Studies such as these should be expanded to NSCLC patients based on similar estrogenic influences on the lung TME.

## Conclusions

While it has long been established that lung tumor cells can be regulated by E2, the role of E2 in the regulation of lung tumor associated stromal and immune cells is now emerging. Estrogen signaling already has a known role in autoimmunity and ERs and aromatase are expressed across multiple immune cell populations and affect their regulation. Differences in the immune system between men and women and the impact of sex hormones in anti-cancer immunity could explain, to an extent, the sex-related disparities in NSCLC incidence, presentation and prognosis. Despite the recent reports on sex-based differences in the use of cancer immunotherapy in NSCLC patients, current knowledge about the effect of sex hormones on tumor immune regulation in the context of lung cancer is still in its infancy. A plethora of literature exists in other tumor types, mainly breast cancer, that E2 orchestrates many immunosuppressive effects on immune cells in the TME. Unraveling the role of E2 in lung tumor immune suppression carries important implications for clinical translation and would provide the rationale for testing combinations of hormonal blockers with current immunotherapy approaches. The immunosuppressive properties of E2 are critical to further evaluate in lung cancer because of the importance of immunotherapy in this disease and the data showing that females do not derive the same benefit to immunotherapy in lung cancer compared to men.

Enhancement of current efficacy rates of ICIs and expansion of its success rate are imperative. It will be important to identify appropriate patient subsets who will benefit from estrogen targeted therapies combined with ICIs. Tobacco smoke exposure causes lung cancer though enhanced inflammation and increased infiltrating immune cells. Increased tobacco exposure may therefore promote up-regulation of an autocrine E2 signaling loop in infiltrating ER and aromatase positive immune cell types in the lung TME, which may contribute to lung cancer progression. Estrogen targeting agents may therefore be especially effective in those patients with underlying inflammatory conditions such as chronic obstructive pulmonary disease or emphysema, who already show heightened immunosuppression. Since immune responses are also observed in very early lung premalignancies, hormonal modulation may play a role in the prevention setting as well (178, 179). As an increased focus is being placed on the interaction between tumor cells and the cells within the TME, the role of estrogen within this context provides a unique perspective that can fit within this complex and constantly changing paradigm. These studies provide potential new strategies for sex-based immunotherapeutic precision medicine approaches for lung cancer and future preclinical and clinical studies should be designed appropriately to take into account sex-related differences, including data analysis by sex and menopausal status. Additionally, antiestrogens, or aromatase inhibitors have few interactions and overlapping toxicities with ICIs and would offer a rational combination approach offering reduced toxicity compared to immunotherapy combinations currently being evaluated, delivering durable responses in a sizable fraction of NSCLC patients.

## Author Contributions

TS prepared the manuscript and figure. LS provided essential input on content and organization and critically edited the manuscript. TB provided vital immunological information and review of content.

### Conflict of Interest

The authors declare that the research was conducted in the absence of any commercial or financial relationships that could be construed as a potential conflict of interest.
